# *Listeria monocytogenes* Infection in Macrophages Induces Vacuolar-Dependent Host miRNA Response

**DOI:** 10.1371/journal.pone.0027435

**Published:** 2011-11-17

**Authors:** Anna K. D. Schnitger, Alzbeta Machova, Roman Ulrich Mueller, Ariadne Androulidaki, Bernhard Schermer, Manolis Pasparakis, Martin Krönke, Nikoletta Papadopoulou

**Affiliations:** 1 Institute for Medical Microbiology, Immunology and Hygiene, University of Cologne, Cologne, Germany; 2 Department of Medicine, University of Cologne, Cologne, Germany; 3 Institute of Genetics, University of Cologne, Cologne, Germany; University of Birmingham, United Kingdom

## Abstract

*Listeria monocytogenes* is a Gram-positive facultative intracellular pathogen, causing serious illness in immunocompromised individuals and pregnant women. Upon detection by macrophages, which are key players of the innate immune response against infection, *L. monocytogenes* induces specific host cell responses which need to be tightly controlled at transcriptional and post-transcriptional levels. Here, we ask whether and how host miRNAs, which represent an important mechanism of post-transcriptional regulation in a wide array of biological processes, are altered by a model pathogen upon live infection of murine bone marrow derived macrophages. We first report that *L. monocytogenes* subverts the host genome-wide miRNA profile of macrophages *in vitro*. Specifically, we show that miR-155, miR-146a, miR-125a-3p/5p and miR-149 were amongst the most significantly regulated miRNAs in infected macrophages. Strikingly, these miRNAs were highly upregulated upon infection with the Listeriolysin-deficient *L. monocytogenes* mutant *Δhl*y, that cannot escape from the phagosome thus representing a vacuolar-contained infection. The vacuolar miRNA response was significantly reduced in macrophages deficient for MyD88. In addition, miR-146a and miR-125a-3p/5p were regulated at transcriptional levels upon infection, and miR-125a-3p/5p were found to be TLR2 responsive. Furthermore, miR-155 transactivation in infection was regulated by NF-κB p65, while miR-146a and miR-125a-3p/5p expression was unaffected in p65-deficient primary macrophages upon *L. monocytogenes* infection. Our results demonstrate that *L. monocytogenes* promotes significant changes in the miRNA expression profile in macrophages, and reveal a vacuolar-dependent miRNA signature, listeriolysin-independent and MyD88-dependent. These miRNAs are predicted to target immune genes and are therefore most likely involved in regulation of the macrophage innate immune response against infection at post-transcriptional levels.

## Introduction

*Listeria monocytogenes* is a Gram positive facultative intracellular bacterium that has been used as a model pathogen to study host-pathogen interactions [1]. This opportunistic food-borne pathogen causes serious illness to immunocompromised individuals, pregnant women and the developing foetus [2]. To establish an infection in a host organism, the bacterium must first overcome the barriers of the innate immune system. Macrophages are professional phagocytes which provide a first line of innate immune defence against invading pathogens. Detection of *L. monocytogenes* by macrophages at the cell surface, within phagosomes or the cytosol triggers distinct host cell transcriptional responses via pattern recognition receptors (PRR) [3]–[4]. Signalling via the so-called vacuolar/Toll-like receptor (TLR) - and the cytosolic/nuclear oligomerisation domain (NOD)-like receptor (NLR) - dependent pathways lead to mitogen-activated protein kinase (MAPK), nuclear factor kappa B (NF-κB) and Interferon Regulatory Factor-3 (IRF3) activation [5]. As a consequence, numerous proinflammatory mediators and other molecules are expressed that further instruct elicitation of antigen-specific acquired immunity and clearance of infection.

To prevent excessive and inappropriate activation of the immune system upon infection with an intracellular pathogen, the host cellular pathways need to be tightly regulated. MicroRNAs (miRNAs) are significant modulators of the immune response that function at post-transcriptional levels [6]. Binding of miRNAs to partially complementary sequences in the 3′ untranslated region (3′ UTR) of their respective protein coding mRNA targets, leads to transcript degradation or translational inhibition [7]–[8]. These small (21–24 nt) non-coding RNAs derive from intergenic sequences, exons or introns of primary transcripts, termed pri-miRNAs, generated mainly by RNA polymerase II [9]. In the nucleus, RNase III Drosha recognizes hairpin structures within the pri-miRNA and generates the precursor miRNA (pre-miR) of approx 60 nt length [10]. Pre-miRs are exported from the nucleus and are further processed in the cytoplasm to the mature miRNA by the RNase III Dicer [11]–[12]. Usually the sense (5p) strand of the mature miRNA is incorporated into the RNA-induced silencing complex (RISC), while the antisense (3p) strand is degraded [13]–[14]. In animals, complementary binding of miRNAs to their mRNA targets is mostly restricted to a 6–9 nt long seed region, which is located at the 5′ end of the miRNA.

Recognition of PAMPs by PRRs of immune cells results in expression of distinct subsets of miRNAs. PRR-triggered miRNAs are thought to target and negatively regulate activated signalling cascade components. In particular, it has been proposed that miR-146a and miR-155 negatively regulate lipopolysaccharide (LPS)-induced TLR4 signalling in myeloid cells with NF-κB transcription factor involved in regulating miR-146a expression [15]–[16]. These miRNAs target protein-coding genes involved in receptor-induced signalling such as TNF receptor-associated factor 6 (TRAF6), IL-1 receptor-associated kinase (IRAK) 1 and IRAK2 by miR-146a [15], [17], or Fas-associated death domain (FADD) and IκB kinase epsilon (IKKε) by miR-155 [18], [19]. In addition, let-7e and miR-155 were found to target TLR4 and SOCS1, respectively, in response to LPS, regulated by Akt1 in macrophages [20]. In infection, miR-155 negatively regulates the epithelial cell response upon infection with Gram-negative bacterium *Helicobacter pylori* [19], while in macrophages infected with another Gram-negative bacterium *Francisella tularensis*, miR-155 expression resulted in enhanced pro-inflammatory response [21]. Similarly, infections with the protozoan parasite *Cryptosporidium parvum* in epithelial cells [22] or detection of heat-killed *Candida albicans* by macrophages [23], lead to alterations of host miRNA profile, most likely involved in immunoregulation. Furthermore, analysis of the host miRNA profile upon *Salmonella Typhimurium* infection also identified the let-7 family of miRNAs as regulators of the inflammatory response in both epithelial cells and macrophages [24]. More recently, it was shown that miR-29 targets interferon (IFN)-γ production by natural killer (NK) cells, CD4+ T cells and CD8+ T cells during systemic infection with intracellular bacteria in mice [25]. However, it is unclear whether infection with any Gram-positive intracellular bacteria can subvert the genome-wide miRNA profile of macrophages.

To address this question, we employed the model pathogen *L. monocytogenes* and first determined whether the host genome-wide miRNA profile is altered upon infection of primary murine macrophages. We found that miR-155, miR-146a, miR-125a-3p/5p and miR-149 were amongst the most significantly regulated miRNAs in infected macrophages. To precisely dissect at which stage of infection and via which host pathways *Listeria* may promote induction of these miRNAs, we used genetically modified *L. monocytogenes* strains, to independently activate the extracellular/vacuolar response, and compare it to wild type *Listeria* that gain access to the cytosol and thus elicits both vacuolar and cytosolic immune responses in macrophages. To accurately identify the host pathways potentially involved in regulation of *Listeria*-induced miRNAs, we directly compared primary macrophages deficient for MyD88 (MyD88^−/−^), which transmits most TLR signals, or NF-κB p65 (p65^MYEL^ KO), which transcriptionally regulates the inflammatory response upon *Listeria* infection, to wild type cells. We have identified miR-155, miR-146a, miR-125a-3p/5p and miR-149 as highly responsive elements of the vacuolar host response, differentially regulated by inflammatory mediators in macrophages.

## Results

### *L. monocytogenes* infection in primary macrophages induces significant host miRNA expression

To determine the impact of *L. monocytogenes* infection in the global miRNA profile of macrophages, we performed parallel profiling of 585 miRNAs using TaqMan Rodent miRNA Arrays A and B (v2.0) on total RNA from bone marrow derived macrophages (BMDM) infected for 6 h with a calculated multiplicity of infection (MOI) 10 compared to non-infected cells, from three biological replicates. Using the Applied Biosystems (ABI) RQ Manager 1.2 and DataAssist v2.0 software for analyses, we comprehensively identified 385 miRNAs that were expressed in our samples, of which 13 were significantly (≥1.5 fold, *P* value≤0.05) up-regulated at 6 h post-infection (pi; Table 1 and Figure S1). Interestingly, *Listeria*-induced miRNAs included miR-155, miR-146a and miR-147, which are known modulators of the inflammatory response upon TLR ligation in macrophages [15]–[16], [26]. Additionally, two recently described miRNAs with unknown function in infected macrophages, namely miR-125a-3p and miR-125a-5p, and a newly described miRNA, namely miR-149, were significantly up-regulated in *Listeria*-infected BMDMs. Surprisingly, no miRNAs were found to be significantly down-regulated in infected compared to non-infected macrophages (Figure S1).

**Table 1:** Significant miRNA expression upon *L. monocytogenes* infection in Bone Marrow Derived Macrophages.

microRNA	Fold change	*P* value
*Array A*		
mmu-miR-146a	2.56	0.0005
mmu-miR-147	3.15	0.0011
mmu-miR-191	1.75	0.0073
mmu-miR-125a-5p	1.76	0.0096
mmu-miR-132	1.54	0.0101
mmu-miR-497	2.27	0.0225
mmu-miR-155	11.57	0.0146
mmu-miR-125a-3p	5.38	0.0263
mmu-miR-200c	2.31	0.0435
mmu-miR-139-5p	1.82	0.0489
*Array B*		
mmu-miR-455*	3.2066	0.0166
mmu-miR-149	2.0487	0.0257
mmu-miR-29b*	2.9698	0.025

Genome-wide profiling was performed by Taqman Rodent miRNA Arrays A and B v2.0 (ABI, Life Technologies).

**Figure 1:**
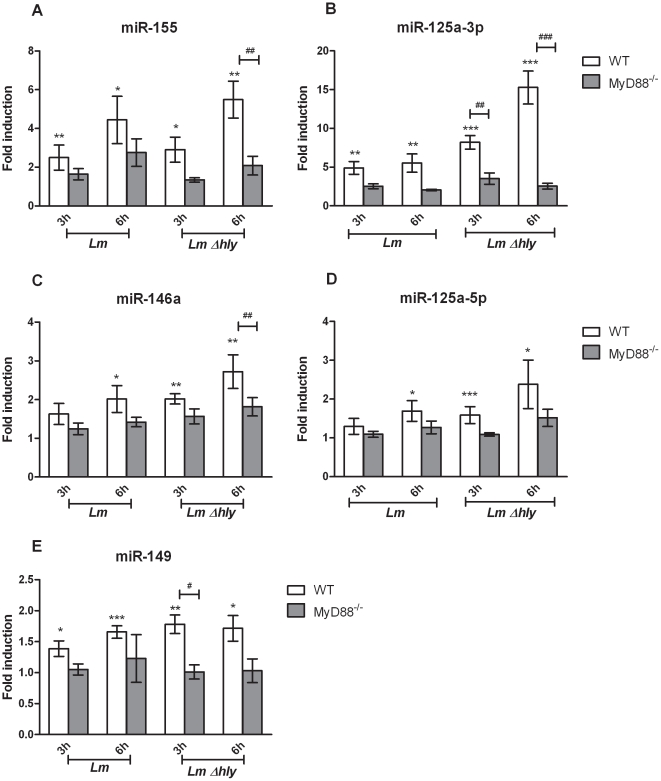
Regulation of *L. monocytogenes*-induced miRNAs in macrophages. Bone marrow derived macrophages (BMDMs) from wild type (WT; n = 4) and MyD88^−/−^ (n = 4) mice were infected (MOI 10) with *L. monocytogenes* (*Lm*) and the LLO-deficient mutant *Δhly* (*Lm Δhly*) for 3 h and 6 h. Total RNA was extracted and the expression levels of miR-155 (A), miR-146a (B), miR-125a-3p (C), miR-125a-5p (D) and miR-149 (E) were quantified by RT-qPCR using TaqMan miRNA assays. Data was normalized to the endogenous control sno202 and fold changes of miRNA expression relative to the non-infected control of its own genotype was calculated by the 2^−ΔΔCT^ method. Data represents the mean ± SEM from three to four biological replicates. Statistical significance of miRNA expression between infected and non infected WT BMDMs was determined by the Student *t*-test; * *P*<0.05; ** *P*<0.01; *** *P*<0.001. Statistical significance of miRNA induction in infection between WT and MyD88^−/−^ BMDMs was determined by two-way ANOVA test; # *P*<0.05, ## *P*<0.01, ### *P*<0.001.

To validate the accuracy of our array results and study further the regulation of these miRNAs upon infection, we generated four new sets of experiments, each including: two timepoints of infection with two types of bacteria (MOI 10) in two different sorts of BMDMs. These samples were subsequently used to quantify miRNA expression by real time quantitative PCR (RT-qPCR) using Taqman assays. The results from these experiments validate that miR-155 and miR-125a-3p were significantly upregulated at 6 h pi by approximately 5-fold (Figure 1A–B). These two miRNAs represent the highest regulated among the five *Listeria*-induced miRNAs. At 6 h pi miR-146a, miR-125a-5p and miR-149 were significantly induced by 1.5–2.0 folds (Figure 1C–E). Interestingly, miR-125a-3p and miR-149 were also significantly up-regulated as early as 3 h pi (Figure 1B and 1E). Taken together, our miRNA-array and RT-qPCR data clearly demonstrate that *L. monocytogenes* subverts the host miRNA profile, causing specific miRNAs to be upregulated early upon infection in BMDMs. These results provide the first miRNA signature of a Gram-positive intracellular pathogen in murine primary macrophages.

### *L. monocytogenes* induced miRNA expression is LLO-independent

Upon uptake by macrophages, *L. monocytogenes* is initially found within the phagosomal vacuole before escaping into the cytosol where it replicates. In parallel, a distinct set of genes is transcriptionally activated upon vacuolar *L. monocytogenes* infection, important for intracellular bacteria sensing [4]. Destruction of the phagosomal membrane by *L. monocytogenes* is essential for *in vivo* bacterial virulence and is mediated by the pore-forming haemolysin Listeriolysin-O (LLO), encoded by the *hly* gene [27]. Therefore, deletion of the gene encoding LLO (*Δhly*) renders this mutant unable to enter the host cell cytosol, avirulent and low in immunogenicity [28].

In our study, we employed this mutant (*Δhly-Lm*) as a tool to investigate compartment-specific miRNA-induction in BMDMs at 3 h and 6 h pi, by which timepoints wild type *Lm* are already replicating in the host cytosol, in contrast to *Δhly-Lm* which remain confined in the phagosome (Figure S2). The intracellular growth of both types of bacteria in macrophages is illustrated in Figure S3. Strikingly, quantitative analyses of miR-155, miR-146a, miR-125a-3p, miR-125a-5p and miR-149 revealed that all five miRNAs were significantly upregulated by the vacuole-contained bacteria at both timepoints (Figure 1A–E). Our findings suggest that these miRNAs are induced in macrophages not only upon wild type/cytosolic *Listeria* infection but also upon vacuolar infection i.e. independent of bacterial virulence and access to the host cytosol.

### Vacuolar miRNA response to infection is MyD88-dependent

Downstream signalling of all TLRs (except of TLR3), IL-1 and IL-18 receptors depends on the adaptor molecule MyD88 [29]. In macrophages, the so-called vacuolar transcriptional response upon *L. monocytogenes* infection is MyD88-dependent, while escape of bacteria in the cytosol induces a MyD88-independent/cytosolic transcriptional response [3]–[4], [30]. To accurately demonstrate which type of host response regulates the *L. monocytogenes*-induced miRNA expression in primary macrophages, and further analyse the strong effect of *Δhly-Lm* infection on miRNA induction, we quantified changes in miRNA expression upon infection with wild type *Lm* or *Δhly-Lm* for 3 h and 6 h in MyD88^−/−^ compared to wild type (WT) BMDMs. Interestingly, expression of miR-155, miR-125a-3p, miR-146a and miR-149 was significantly reduced in MyD88^−/−^ compared to WT BMDMs upon infection with *Δhly*-*Lm* (Figure 1A–C and 1E, respectively). The effect of MyD88 ablation in miR-125a-5p induction was moderate (Figure 1D). These findings suggest that *L. monocytogenes* promotes a predominant vacuolar miRNA response that is MyD88-dependent.

We additionally analysed BMDM culture supernatants from the same infections for tumour necrosis factor (TNF) production, a gene that represents induction of the vacuolar response upon *Listeria* infection in macrophages [4]. In agreement to the published data, TNF production was induced upon wild type *Lm* and *Δhly-Lm* infection in WT BMDMs and completely abolished in MyD88^−/−^ BMDM (Figure S4), while bacterial uptake in cells of both genotypes was comparable (Figure S5).

### *L. monocytogenes* infection promotes upregulation of miRNA genes at transcriptional level

MiR-125a-3p and miR-125a-5p represent two opposing strands of the same primary transcript (pri-miR; Figure S6). Nevertheless, *Listeria*-induced expression levels of the two mature miRNAs varied between the two strands by 4.5-folds (Figure 1B and 1D) suggesting differential regulation upon infection. Regulation of mature miRNA expression can occur on the level of transcription as well as post-transcriptionally [15]–[16], [31]. To determine whether *L. monocytogenes* regulates miRNA-125a-3p/5p expression in BMDMs at transcriptional level, we analysed the induction of pri-miR-125a, in parallel to pri-miR-146a, upon 3 h and 6 h pi with wild type *Lm* and *Δhly-Lm*.

**Figure 2:**
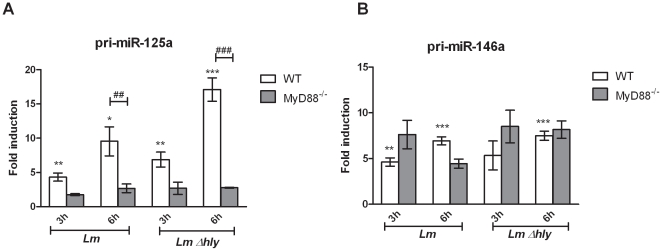
*L. monocytogenes*-induced transcriptional regulation of miR-146a and miR-125a. Bone marrow derived macrophages (BMDMs) were infected (MOI 10) with *L. monocytogenes* (*Lm*) and the LLO-deficient mutant *Δhly* (*Lm Δhly*) for 3 h and 6 h. Total RNA was extracted and the expression levels of primary transcript (pri)-miR-125a (A) and pri-miR-146a (B) were quantified by RT-qPCR using TaqMan assays. Data was normalized to the endogenous control gene HPRT1 and fold changes of miRNA induction in infected compared to control cells of each genotype were calculated by the 2^−ΔΔCT^ method. Data represents the mean ± SEM from three biological replicates. Statistical significance of miRNA expression between infected and non infected WT BMDMs was determined by the Student *t*-test; * *P*<0.05, ** *P*<0.01, *** *P*<0.001. Statistical significance of miRNA induction in infection between WT and MyD88^−/−^ BMDMs was determined by two-way ANOVA test; # *P*<0.05, ## *P*<0.01, ### *P*<0.001.

We found that pri-miR-125a and pri-miR-146a were both upregulated upon cytosolic and vacuolar infections, while pri-miR-125a induction was significantly augmented upon *Δhly-Lm* infection (Figure 2). Furthermore, we compared miRNA transcriptional regulation upon infection of MyD88^−/−^ and WT BMDMs and found that pri-miR-125a upregulation was significantly decreased in infected MyD88^−/−^ BMDM (Figure 2A) suggesting that MyD88 signalling is also required for the upregulation of the primary transcript upon infection, most likely upon immediate recognition of *Listeria* PAMPs extracellularly or in the phagosomal vacuole. In contrast, pri-miR-146a induction was unaffected in MyD88^−/−^ BMDMs compared to WT cells infected by either wild type *Lm* or *Δhly-Lm* (Figure 2B). Our findings suggest that *Listeria* infection in BMDMs regulates both miR-125a-3p/5p and miR-146a at transcriptional levels, the first in a vacuolar/MyD88-dependent manner and the latter in a MyD88-independent manner.

### TLR2-mediates miR-125a-3p and miR-125a-5p upregulation in macrophages

**Figure 3:**
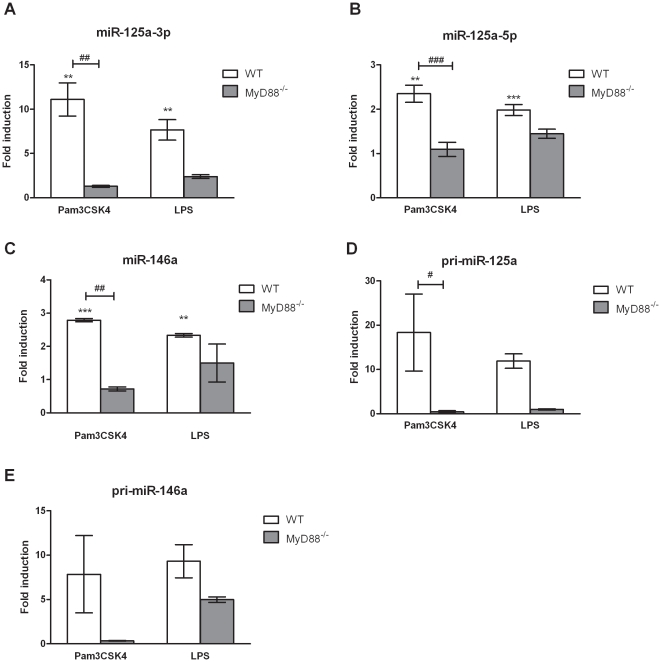
Regulation of miR-125a-3p/5p and miR-146a expression upon TLR2 and TLR4 stimulation. Wild type (WT; n = 3) and MyD88^−/−^ (n = 2) mouse bone marrow derived macrophages (BMDMs) were treated with 1 µg/ml ultra-pure LPS or 2 µg/ml Pam3CSK4 for 6 h. Total RNA was extracted and miRNA expression levels were quantified by RT-qPCR using TaqMan miRNA assays. MiR-125a-3p (A), miR-125a-5p (B) and miR-146a (C) expression in MyD88^−/−^ BMDMs. Pri-miR-125a (D) and pri-miR-146a (E) expression upon TLR stimulation in WT and MyD88^−/−^ BMDMs. Data was normalized to sno202 or HPRT1, endogenous controls for miR or pri-miR expression, respectively, and fold changes were calculated by the 2^−ΔΔCT^ method and expressed relative to non-infected control of each genotype. Data represents the mean ± SEM of at least two biological replicates. Statistical significance of miR/pri-miR induction was determined by the Student *t*-test compared to non infected control (* *P*<0.05, ** *P*<0.01, *** *P*<0.001) or two-way ANOVA between WT and KO cells (# *P*<0.05, ## *P*<0.01, ### *P*<0.001).

Upon infection, *L. monocytogenes*-derived lipoteichonic acids [32] and lipoproteins [33] are recognised by membrane-bound TLR2 receptors which transmit their signals via MyD88 [34]. Activation of TLRs in immune cells results in expression of distinct subsets of miRNAs, including miR-146a [15] and miR-155 [16]. To investigate the response of miR-125a-3p and miR-125a-5p upon receptor-specific activation, we stimulated BMDMs with the TLR2 synthetic ligand Pam3CSK4. Interestingly, our results showed that miR-125a-3p and miR-125a-5p were significantly induced upon Pam3CSK4 treatment in BMDMs, providing the first evidence to support involvement of the TLR2 pathway in the induction of these two miRNAs (Figure 3A–B). Recently, miR-125a-3p and miRNA-125a-5p were reported to be moderately upregulated upon TLR4 ligation by LPS, a major component of the cell wall of Gram-negative bacteria, in BMDMs subjected to microarray analyses (*P* values of 0.095 and 0.066 respectively) [23]. Unlike this study, we used ultra-pure LPS to prevent residual protein impurities influencing the induction attributed to TLR4-signalling, and found that miR-125a-3p and miR-125a-5p were both significantly upregulated upon specific TLR4 ligation (*P*<0.01 and *P*<0.001, respectively) similarly to miR-146a which served as positive control (Figure 3A–C). Most importantly, we compared TLR2- and TLR4-mediated upregulation of miR-125a-3p, miR-125a-5p and miR-146a in BMDMs from WT and MyD88^−/−^ mice, and could show that TLR2-mediated upregulation of all three miRNAs by Pam3CSK4 was significantly decreased in MyD88^−/−^ cells and thus was MyD88-dependent (Figure 3A–C). Compared to WT cells, MyD88^−/−^ BMDMs showed impaired TNF production upon stimulation with Pam3CSK4 and LPS (Figure S2). Notably, TLR4-mediated miRNA expression induced by LPS was not entirely MyD88-dependent, since for all three miRNAs, expression was slightly but not significantly reduced in MyD88^−/−^ compared to WT cells (Figure 3A–C). This is most likely due to the action of the adaptor protein TIR domain containing adaptor inducing interferon beta (TRIF) that transmits MyD88-independent TLR4 signals. Similarly, TLR2 and TLR4 activation led to upregulation of miR-125a and miR-146a primary transcripts in WT BMDMs, while induction of pri-miR-125a was MyD88-dependent (Figure 3D–E). These findings support that miR-125a-3p and miR-125a-5p are new members of the group of miRNAs that are induced upon TLR/MyD88-signalling and underline a role in the vacuolar response of macrophage to infection.

### NF-κB p65 regulates miR-155, but not miR-125a-3p/5p and miR-146a, expression in response to *L. monocytogenes* or LPS

**Figure 4:**
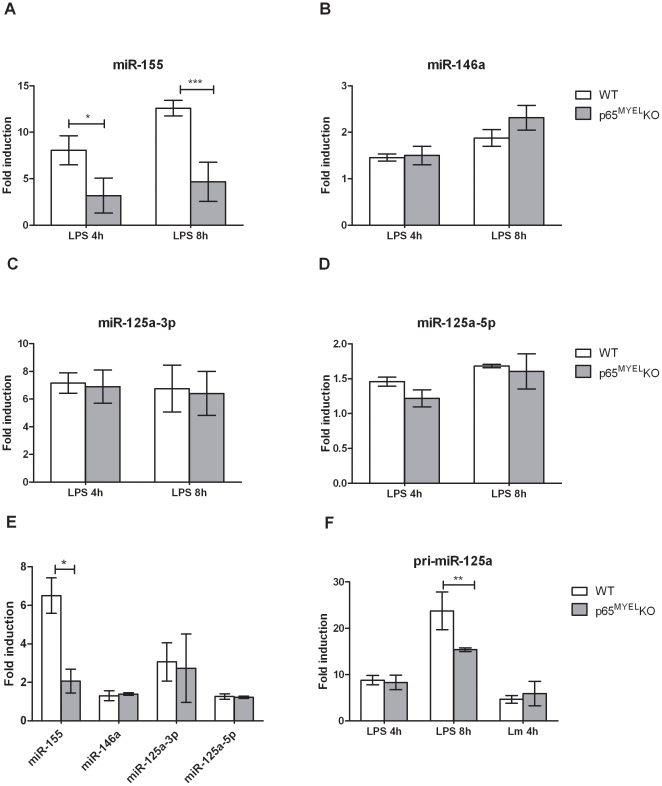
Regulation of miR-125a-3p/5p, miR-146a and miR-155 expression in macrophages by NF-κB p65. Wild type (WT) and p65^MYEL^ KO BMDMs were treated with 100 ng/ml ultra-pure LPS for 4 h and 8 h (A–D, F) or infected (MOI 1) with *L. monocytogenes* (*Lm*) for 4 h (E–F). Total RNA was extracted and the expression levels of miR-125a-3p, miR-125a-5p, miR-146a and miR-155 were quantified by TaqMan miRNA assays. Data was normalized to sno202 or HPRT1, endogenous controls for miR or pri-miR expression, respectively, and fold changes were calculated by the 2^−ΔΔCT^ method and expressed relative to the mock-treated/mock-infected control for each timepoint/condition in each genotype. Data represents the mean ± SEM of at least two independent experiments, including minimum two mice from each genotype. Statistical significance of miR/pri-miR induction between WT and p65^MYEL^ KO BMDMs was determined by two way ANOVA; * *P*<0.05, ** *P*<0.01, *** *P*<0.001.

A potential mechanism for selectively regulating miRNA transcript levels upon infection is via nuclear transcription factors [15], [22], [35]. Activation of NF-κB transcription factors downstream of TLRs is crucial in coordinating the transcriptional response to infection, and various NF-κB subunit knock-out mice show increased susceptibility to *L. monocytogenes* infection [36]. Few studies report that miR-155 and miR-146a have active NF-κB binding sites in their promoter elements [15], [37], or their induction depends on signalling via the IκB kinase (IKK) complex [21]–[23]. We hypothesised that NF-κB p65 is involved in regulating transcription of *L. monocytogenes*-induced miRNAs, and to accurately demonstrate its direct role we compared BMDMs from mice in which p65 is genetically ablated (p65^MYEL^ KO) to WT cells upon mild (MOI 1) infection with *L. monocytogenes* for 4 h or exposure to LPS for 4 h and 8 h. To corroborate the absence of functional p65 we first assessed the response of TNF, a p65-regulated gene. As expected, p65^MYEL^ KO BMDMs showed reduced TNF production upon infection or LPS treatment compared to WT cells (Figure S7). Similarly, miR-155, which possesses two active NF-κB binding sites in the BIC/miR-155 promoter region, was significantly reduced in p65^MYEL^ KO BMDMs compared to WT cells upon LPS treatment and *L. monocytogenes* infection (Figure 4A and 4E), suggesting the involvement of the canonical NF-κB pathway, and specifically of the p65 subunit. Surprisingly, miR-146a expression which was shown to be NF-κB p65 dependent upon LPS treatment in mouse embryonic fibroblasts [38] was unaffected in p65^MYEL^ KO compared to WT BMDMs treated with LPS or infected with *L. monocytogenes* (Figure 4B and 4E). Similarly, miR-125a-3p and miRNA-125a-5p which are predicted to derive from exon1 of a non-coding RNA transcript on chromosome 17 (Ensembl: Ncrna 00085-003) that has several putative NF-κB binding sites in the pri-miR-125a promoter region, was unaffected by genetic ablation of p65 in infected or LPS treated BMDMs (Figure 4C–E). Furthermore, we specifically investigated the pri-miR-125a kinetics and found that only at 8 h after LPS exposure, its expression was significantly reduced in p65^MYEL^ KO compared to WT BMDMs (Figure 4F). Based on *in silico* analyses of the 1.5 kb region upstream of the pri-miR-125a transcriptional start site for transcription factor binding sites, using the Genomatix MatInspector software package, we found that apart of the two putative NF-κB p65 sites, there are also putative NF-κB p50 and IRF binding sites present in the promoter region (Figure S6). These results suggest that regulation of *L. monocytogenes*-induced pri-miR-125a may be propelled by NF-κB p50 or another transcription factor, which is also likely for miR-146a, while miR-155 transactivation upon *L. monocytogenes* infection and LPS treatment in BMDMs is predominately NF-κB p65 dependent.

## Discussion

It is well established that miRNAs are important players of the post-transcriptional mechanism that regulates gene expression in many biological processes, including cell proliferation, differentiation, immunity and tumorigenesis. Host-pathogen interactions must be tightly controlled at all levels to prevent excessive inflammation and spread of infection. It is increasingly appreciated that miRNAs are important immunoregulators; however our knowledge on the role of miRNAs upon bacterial infection is far from comprehensive. Infection of macrophages with one of the best studied Gram positive intracellular pathogens *L. monocytogenes* initiates a sequence of responses at transcriptional and post-transcriptional levels which are vital for host survival. In this model, we first explored whether miRNAs are part of the anti-listerial immune response of macrophages. Our study reveals the *Listeria*-induced miRNA signature at 6 h pi in primary macrophages. Specifically, infection induced significant upregulation of 13 miRNAs, including miR-155, miR-146a, miR-125a-3p/5p and miR-149. To our knowledge, this is the first comprehensive profile of miRNA expression upon Gram-positive bacterial infection. Notably, at 6 h pi, we did not detect significantly downregulated miRNAs in infected BMDMs compared to non-infected control cells. To establish profound down-regulation of miRNAs, longer timepoints may need to be explored [22], [24]–[25] though in that case, the contribution of cytokines and interferons produced by infected macrophages should also be considered when attributing the direct and indirect effects of bacterial infection on the host miRNA profile [16].

Our aim was to investigate potential miRNA induction linked to PRR activation which is vital for innate immune defence against intracellular pathogens.

To validate our array data, we performed quantitative RT-PCR and confirmed that miR-155, miR-146a, miR-125a-3p/5p and miR-149 were significantly increased in macrophages upon early sensing of live bacteria, suggesting that these miRNA genes may be implicated in the inflammatory immune response. MiR-149 has not been characterised before in macrophages upon TLR activation, while miR-125a-3p/5p were only recently reported to be upregulated upon LPS treatment or heat-killed *C. albicans* infection, in monocytes [39] and macrophages [23], respectively. MiR-155 and miR-146a are induced in different cell types upon viral, parasitic, fungal or Gram-negative bacterial infections as well as upon specific TLR ligation and are thought to modulate the inflammatory response [15]–[24], [37].

To determine how *L. monocytogenes* infection promotes host miRNA induction, we differentiated the phases of infection to vacuolar or cytosolic which are characterized by distinct transcriptional profiles in macrophages [3]–[4]. To accomplish this, we employed two different types of *L. monocytogenes* EGDe bacteria, the wild type and its isogenic mutant *Δhly* [28] which is confined to the phagosome and therefore does not induce cytosolic immune signalling. Interestingly, like wild type infection, vacuolar/*Δhly-Lm* infection caused significant upregulation of all five miRNAs, suggesting that the haemolytic action of LLO and therefore the cytosolic surveillance pathways are not implicated in the miRNA response of macrophages to infection. To investigate whether this response is regulated via the TLR signalling pathway and establish the effect of vacuolar infection to the host miRNA response, we compared miRNA induction between WT and MyD88^−/−^ BMDMs, upon *wt-Lm* and *Δhly-Lm* infections. Our data shows that miR-155, miR-125a-3p, miR-146a and miR-149 response is MyD88-dependent upon *Δhly-Lm* infection, suggesting that these miRNAs are among the early-response genes expressed in macrophages upon phagosomal detection of *Listeria* PAMPs. These data also provide the first evidence on miR-149 and miR-125a-3p regulation by MyD88.

From all *Listeria*-induced miRNAs, we were particularly attracted to miRNA-125a-3p and miRNA-125a-5p, because they represent the two strands of the same miRNA duplex, and unlike their trend of induction upon LPS treatment or heat-killed *C. albicans* infection [23], upregulation upon *Listeria* infection varied significantly between the two miRNA strands. *Listeria* promotes miR-125a gene expression upon MyD88 signalling at the transcriptional level which was comparable to the induction and regulation of the mature miR-125a-3p. In contrast, miR-125a-5p expression during both *wt-* and *Δhly-Lm* infections was moderately affected by MyD88 deletion in BMDMs. Based on the mechanism proposed by Schwarz et al. [13], miRNA 3p and 5p strand accumulation depends on free energy properties of miRNA duplexes, and can be developmentally regulated in specific cell lineages [40]. It is tempting to speculate that microbial infection may also influence the mechanisms that regulate mature miRNA abundance and strand stability in activated macrophages, providing an additional element to the proposed mechanism, perhaps depending on miRNA target abundance and the state of the infected cell. Furthermore, recent evidence on miRNA-125a-3p/5p in lung carcinoma suggests that the two strands exhibit opposing functions in regulating invasive and metastatic capabilities of lung cancer cells [41]. Although their role in infection is still unresolved, we clearly show that miR-125a-3p/5p, alongside miR-155 and miR-146a, are part of the early innate immune response of macrophages to *Listeria* infection. The majority of the targets for miR-155 and miR-146a are still unknown [15], [17]–[19], [21], [42]–[44], while the targets for miR-125a-3p/5p and miR-149 have not yet been explored. We carried out computational analyses by *TargetScanMouse 5.1* software as previously described [22], [45], and identified a variety of immune-related putative mRNA targets of all five *L. monocytogenes*-induced miRNAs (Table S1). MiR-125a-3p and miR-125a-5p potentially target the interleukin-1 receptor 1 (IL-1 R1), and the IL-6 receptor (IL-6 R), respectively. MiR-125a-5p shares the same 5′-UTR sequence (seed region) with miR-125b and may therefore target the same mRNAs. In murine macrophages, miR-125b was shown to be induced by LPS and to target TNFα [18] and in H69 epithelial cells it was upregulated upon *C. parvum* infection via NF-κB [22]. However, miR-125b was not upregulated in our arrays, which is not surprising since the two miRNAs derive from transcripts located on different chromosomal locations, and are probably regulated differently in BMDMs infected with *L. monocytogenes* compared to other types of infection.

The recognition of *Listeria* PAMPs by TLR2 in macrophages is well established [32]–[34]. MiR-155 and miR-146a have been shown to be induced upon ligation of TLR2 and TLR4 [15]–[16]. In contrast, the role of TLR2 on miR-125a-3p/5p induction and regulation has not been recognised. We have demonstrated that miR-125a-3p/5p were upregulated upon TLR2 activation, which turned out to be MyD88-dependent. The TLR2-MyD88-dependent induction of miR-125a-3p/5p was found to be regulated at transcriptional levels. Furthermore, miR-125a-3p/5p were significantly upregulated upon LPS stimulation, although the requirement of MyD88 at both mature and primary-transcript levels was less evident, possibly due to involvement of TLR4-TRIF-mediated signalling, that is also induced upon LPS treatment in BMDMs [38]. Recent evidence suggests that miR-125a-5p is involved in the atherosclerotic response of monocytes/macrophages since its expression was induced by ox-LDL stimulation of THP-1 cells while its down-regulation lead to reduced lipid uptake and inflammatory cytokine secretion, including IL-6, TNF- α, and TGF-β [46]. Therefore, miR-125a gene is likely to have a broad role in modulating the inflammatory response of myeloid cells.

A potential mechanism for selectively regulating miRNA transcript levels upon immune stimuli is via nuclear transcription factors [22], [35], [47]. Promoter binding by NF-κB or pharmacological inhibition of IKK signalling was used previously to show regulation of miR-146a and miR-155 expression upon Gram-negative bacterial infections or LPS stimulation in several independent studies [15], [21], [23], [37]–[38]. Similarly, during *C. parvum* infection in epithelial cells several miRNA genes were activated upon NF-κB p65 binding to their promoter regions [22]. NF-κB p65 is a member of the NF- κB family of transcription factors, crucial for induction of proinflammatory gene transcription upon *Listeria* infection [36]. We used NF-κB p65 deficient BMDMs to investigate miR-125a-3p/5p regulation upon *L. monocytogenes* infection or LPS treatment, in parallel to miR-146a and miR-155. Unlike miR-155, miR-125a-3p/5p and miR-146a expression were unaffected in p65^MYEL^ KO compared to WT BMDMs infected with *L. monocytogenes* or treated with LPS. The expression of pri-miR-125a following LPS challenge, but not upon *L. monocytogenes* infection, was significantly decreased in p65^MYEL^ KO compared to WT BMDMs, which indicates a specific requirement for p65 in miR-125a-3p/5p gene transcription. In agreement to this observation, IKKβ was reported to play a role in miR-125a transcription upon fungal PAMP- or LPS-treated macrophages [23] suggesting that NF-κB regulates miR-125a transcription in specific conditions and types of infection. Similarly, we observed increased TNF production upon *wt* and *Δhly-Lm* infections, which coincides with increased miR-155 expression, both of which were regulated by NF-κB p65. Perhaps *Listeria*-induced miR-155 is involved in regulating TNFα mRNA stability in BMDMs. Finally, infection with the Gram-negative bacterium *Francisella novicida*, but not *Francisella tularensis*, induced miR-155 expression in human monocytes [21]. Similarly to *F. novicida*, *L. monocytogenes Δhly* cannot escape from the phagosome in the cytoplasm of the host cell, unlike the virulent strain *F. tularensis* which led to a significantly lower miR-155 expression. Thus, it is possible that miR-155 is a common element of the host innate immune response to phagosome-confined bacteria following activation of TLR2/MyD88 signalling.

In summary, our study reveals the first genome-wide *Listeria*-induced miRNA profile in primary murine macrophages. Our findings show significant upregulation of specific miRNAs upon infection. Most importantly, expression of these miRNAs was augmented during the vacuolar host response, in a MyD88-dependent manner, and differentially regulated by NF-κB p65, suggesting that they are part of the early innate immune response of macrophages to bacterial infection. The *Listeria*-induced miRNA signature is composed of the well established in immune regulation miR-155 and mR-146a, as well as the newly detected miR-149 and the miR-125a-3p/5p duplex, all of which are predicted to target important immune-related genes. Furthermore, this study signifies the miRNA host response upon Gram positive intracellular bacterial infection in macrophages providing new aspects of regulation in host-pathogen interactions, at post-transcriptional levels.

## Materials and Methods

### Ethics Statement

All animal procedures were conducted in accordance with European (EU directive 86/609/EEC), national (TierSchG), and institutional guidelines and protocols of the University of Cologne, and were approved by local governmental authorities (Landesamt für Natur, Umwelt und Verbraucherschutz Nordrhein-Westfalen) under the licenses 8.87-50.10.45.08.219 and 8.87-50.10.37.09.242.

### BMDM primary cell culture

BMDMs were isolated from the femurs and tibias of WT and KO C57BL/6 mice, 8–12 weeks old. After red blood cell lysis, cells were plated in RPMI supplemented with 10% FCS, 15% L929-conditioned medium as a source of the macrophage colony-stimulating factor (M-CSF), 100 units/ml penicillin and streptomycin, 2 mM glutamine, 1 mM sodium glutamate and HEPES. Cells were cultured for 7–8 days in bacterial dishes in a humidified incubator with 5% CO_2_ at 37°C and differentiated BMDMs were used for experiments on day 8–10. Over 95% of these cells were F4/80 and CD11b double positive as determined by FACS analyses (Figure S8).

### Reagents

Ultra-pure LPS from *E. coli* serotype EH100 (Ra) TLR-grade was obtained from Alexis Biochemicals and Pam3CSK4 was purchased from Invivogen. TNF production was measured in the culture supernatants using an ELISA kit (R&D Systems). TRITC-conjugated Phalloidin and goat anti-rabbit IgG FITC conjugates were purchased from SIGMA; polyclonal antibody against *L. monocytogenes* was purchased from Acris. Mmu-miRNA detection assays were obtained from Applied Biosystems (ABI, Life Technologies): sno202 (assay ID: TM 001232); miR-125a-3p (assay ID: TM 002199); miR-125a-5p (assay ID: TM 002198); miR-146a (assay ID: TM 000468); miR-149 (assay ID: TM 002255); miR-155 (assay ID: TM 002571); pri-mir-146a (Mm03306349); pri-mir-125a (Mm03306233); HPRT1(Mm01545399_m1).

### Mice

All mice were kept under specific pathogen-free conditions. WT C57BL/6 mice were purchased from Charles River. MyD88^−/−^ mice, on a C57BL/6 genetic background, were previously described by Adachi *et al.*, 1998 [48]. The C57BL/6 Mx1Cre mice were crossed with p65^FL/FL^ transgenic mice to generate p65^MYEL^ KO upon systemic administration of polyinosine-polycytidylic acid (polyI:C).

### Bacteria

*L. monocytogenes* EGDe wild-type, serotype 1/2a, and the isogenic deletion mutant *L. monocytogenes* EGDe *Δhly* [28] were kindly provided by E. Domann and T. Chakraborty (Justus-Liebig-University, Giessen, Germany). Bacteria were grown from single colonies in BHI medium at 37°C with shaking, and were harvested during mid-log phase. Bacteria were washed three times in PBS and the optical density of the culture was measured at 600 nm to calculate the required inoculum for infections.

### Bacterial infections

One day prior to infection, BMDMs were seeded in cell culture dishes in complete RPMI medium without antibiotics. Cells were infected with normal mouse serum (NMS)-opsonised bacteria at a multiplicity of infection (MOI) of 1 or 10. At 30 min pi, non-infected control cells and infected cells were washed 4 times with PBS before the addition of fresh medium. Samples were taken at 3 h and 6 h pi. Additionally, at 1 h, 3 h and 6 h after infections in BMDMs, cells were lysed in 0.1% Triton-X100 in H2O. Serial dilutions (10^−2^–10^−6^) were plated on blood agar plates overnight and colony forming units were counted the next day, to determine the rate of bacterial growth.

### RNA isolation

Total RNA from BMDMs was isolated with TRIzol according to the manufacturer's protocol (Invitrogen, Life Technologies). The quantity of total RNA was measured at the *NanoVue* spectrophotometer (GE Healthcare) and quality was determined by automated gel electrophoresis on the Experion system (Bio-Rad). Prior to pri-miR assays, RNA was DNase I-treated using the Ambion TURBO DNA-free kit (Ambion, Life Technologies).

### TaqMan Rodent miRNA Arrays

TaqMan Rodent miRNA Arrays A and B (v2.0) were used for genome-wide profiling according to the Sanger miRBase v10, in non-infected and *L. monocytogenes*-infected BMDMs (6 h pi, MOI 10) from three independent experiments. Total RNA (800 ng) was reverse transcribed using miRNA-specific Megaplex RT Primer-Pools A and B with the TaqMan Reverse Transcription Kit (ABI, Life technologies). The respective RT reactions were distributed into the allocated ports of the preloaded miRNA Array cards A and B and Taqman was performed for profiling 585 miRNAs, including controls, according to the manufacturer's instructions, on an ABI 7900HT sequence detection system. ABI RQ Manager 1.2 and the DataAssist v2.0 software (Life Technologies) were used to analyse the data. Data was normalized to the endogenous controls mU6 and sno202, and miRNAs with a Ct value ≤35 were included in the analysis. MiRNA expression fold changes were calculated by the 2^−ΔΔCT^ method [49], and Student *t*-test was performed to determine significance. MiRNAs with a fold change ≥1.5 and with a *P* value ≤0.05 were classified as significantly regulated.

### TaqMan pri- and miRNA assays

Mature miRNA expression was quantified using TaqMan microRNA assays (ABI, Life Technologies). Total RNA (10 ng) was reversed transcribed using miRNA specific primers and the TaqMan Reverse Transcription Kit (ABI, Life Technologies). TaqMan miRNA assays were performed on a Roche LC480 LightCycler, using the TaqMan Universal PCR Master Mix (ABI, Life Technologies) and analyzed with the LC480 analysis software (Roche). For quantification of pri-miR-miRNA levels, DNase I-treated total RNA (500 ng) was reverse transcribed using Invitrogen SuperScript II reverse transcriptase (Invitrogen, Life Technologies). The TaqMan pri-miR assays were performed according to the manufacturer's instructions (ABI, Life Technologies). Relative fold changes of gene expression were determined with the 2^−ΔΔCT^ method [49]. Values were normalized to the endogenous controls, sno202 for mature miRNAs and Hypoxanthin-Guanin-Phophoribosyltransferase 1 (HPRT1) for pri-miRs. Statistical significance was determined by Student *t*-test or two-way ANOVA.

## Supporting Information

Figure S1 ***L. monocytogenes*** **infection in primary macrophages induces significant host miRNA expression.** TaqMan Rodent miRNA Arrays A and B (v2.0) were used to profile 585 miRNAs, including controls, in non-infected and *L. monocytogenes*-infected BMDMs at 6 h, MOI 10, from three independent experiments. Total RNA (800 ng) was reverse transcribed using miRNA-specific Megaplex RT Primer-Pools A and B with the TaqMan Reverse Transcription Kit (Life Technologies). Data was normalized to the endogenous controls mU6 and sno202 using the ABI RQ Manager 1.2 and the DataAssist v2.0 software (Life Technologies). MiRNAs with a Ct value ≤35 were included in the analysis. Fold changes ≥1.5 calculated by 2^−ΔΔCT^ method, and *P* values≤0.05 determined by Student *t*-test, were used to identify significantly regulated miRNAs.
(TIF)

Figure S2 **Immunofluorescence staining of macrophages infected with** ***L. monocytogenes*** **(** ***Lm*** **).** Bone marrow derived macrophages (BMDMs) were infected (MOI 10) with *Lm* (A and C) or the LLO-deficient mutant *Δhly* (B and D) for 3 h (A and B) and 6 h (C and D). Cells were fixed, permeabilized and stained with TRITC-phalloidin antibody against filamentous actin (red), anti-*Listeria* primary antibody with FITC-conjugated goat anti-rabbit secondary antibody, to detect bacteria (green). Cell nuclei were stained with DAPI (blue). Immunomicrographs were taken with a 40× objective.
(TIF)

Figure S3 **Growth curves of** ***L. monocytogenes*** **(** ***Lm*** **) wild type (wt) and** ***Δhly*** **mutant in macrophages.** Cells were infected for 30 min and samples were collected at indicated timepoints post infection. Cells were lysed in 0.1% Triton-X100 in H2O and serial dilutions (10^−2^–10^−6^) were plated on blood agar plates overnight. Colony forming units were counted the next day and cfu/ml were plotted from 2–3 replicates.
(TIF)

Figure S4 **TNF production in wild type (WT) and MyD88^−/−^ primary macrophages.** Cells were treated for 6 h with 2 µg/ml Pam3CSK4 or 1 µg/ml LPS; or infected (MOI 10) for 3 h and 6 h with *L. monocytogenes* (*Lm*) or the LLO-deficient mutant *Δhly* (*Lm Δhly*). TNF (pg/ml) production was measured in the culture supernatant collected from 1×10^6^ cells/ml by ELISA. Data represents the mean values ± SEM from two biological replicates.
(TIF)

Figure S5 **Immunofluorescence staining of macrophages infected with** ***L. monocytogenes*** **(** ***Lm*** **).** Wild type (WT; A and C) or MyD88^−/−^ (B and D) bone marrow derived macrophages (BMDMs) were infected with *Lm* MOI 10, for 3 h (A and B) or 6 h (C and D). Cells were then fixed, permeabilized and stained with TRITC-phalloidin antibody against filamentous actin (red), anti-*Listeria* primary antibody with FITC-conjugated goat anti-rabbit secondary antibody, to detect bacteria (green). Cell nuclei were stained with DAPI (blue). Immunomicrographs were taken with a 40× objective.
(TIF)

Figure S6 **Schematic diagram of miR-125a genomic locus on mouse chromosome 17.** Putative binding sites of NF-κB and IRF3/7 are shown (boxes) within the 1.5 kb upstream region of the pri-miR-125a transcriptional start site. Pre-miR-125a matures in the cytoplasm giving rise to mature miR-125a-5p and miR-125a-3p.
(TIF)

Figure S7 **TNF production in wild type and p65^MYEL^KO macrophages.** WT (n = 2) and p65^MYEL^KO (n = 2) bone marrow derived macrophages (BMDMs) were infected with *L. monocytogenes* (*Lm*) MOI 1 for 4 h, or treated with 100 ng/ml LPS for 4 h and 8 h. TNF (pg/ml) production was measured in the culture supernatant collected from 1×10^6^ cells/ml by ELISA. Data represents the mean values ± SEM from one experiment.
(TIF)

Figure S8 **Characterisation of differentiated bone marrow derived macrophages (BMDMs) by FACS.** BMDMs from days 7–8 of differentiation were stained with F4/80 (eBiosciences) and/or CD11b (BD) antibodies and analysed at the FACS Calibur. Quadrant statistics was performed using Cell Quest (BD).
(TIF)

Table S1 **Predicted and confirmed miRNA targets selected on the basis of their potential or known involvement in the anti-bacterial immune response of macrophages.** Putative targets were predicted using TargetScan 5.1. Confirmed targets were selected from the cited studies.
(DOCX)
