# Supplementary figures and images for: Listeria monocytogenes Infection in Macrophages Induces Vacuolar-Dependent Host miRNA Response
Source: PLoS One. 2011 Nov 17;6(11):e27435. doi: 10.1371/journal.pone.0027435 (PMC3219661; doi:10.1371/journal.pone.0027435)

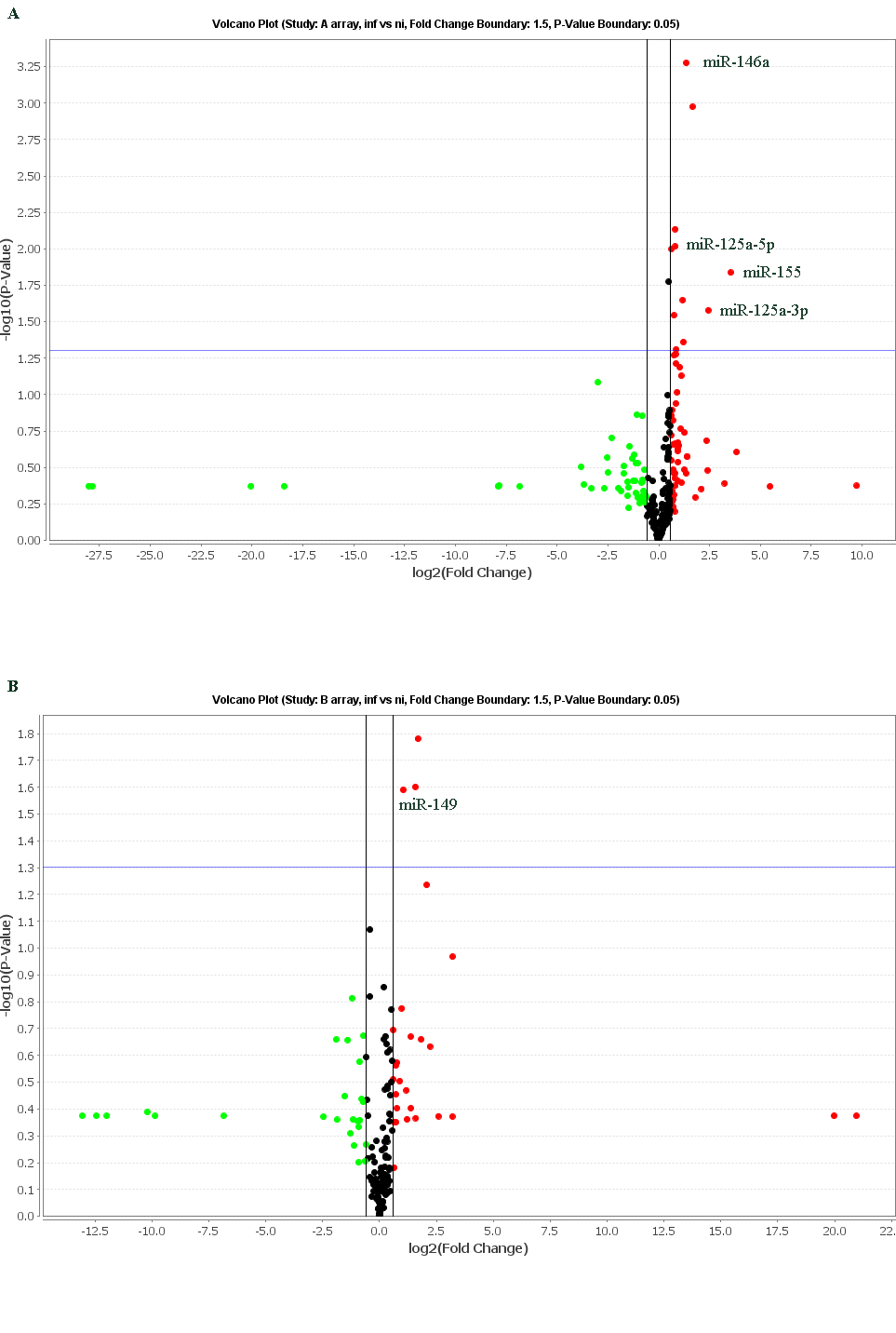

Supplement: Figure S1 — L. monocytogenes infection in primary macrophages induces significant host miRNA expression. TaqMan Rodent miRNA Arrays A and B (v2.0) were used to profile 585 miRNAs, including controls, in non-infected and L. monocytogenes-infected BMDMs at 6 h, MOI 10, from three independent experiments. Total RNA (800 ng) was reverse transcribed using miRNA-specific Megaplex RT Primer-Pools A and B with the TaqMan Reverse Transcription Kit (Life Technologies). Data was normalized to the endogenous controls mU6 and sno202 using the ABI RQ Manager 1.2 and the DataAssist v2.0 software (Life Technologies). MiRNAs with a Ct value ≤35 were included in the analysis. Fold changes ≥1.5 calculated by 2−ΔΔCT method, and P values≤0.05 determined by Student t-test, were used to identify significantly regulated miRNAs. (TIF) [file pone.0027435.s001.tif]

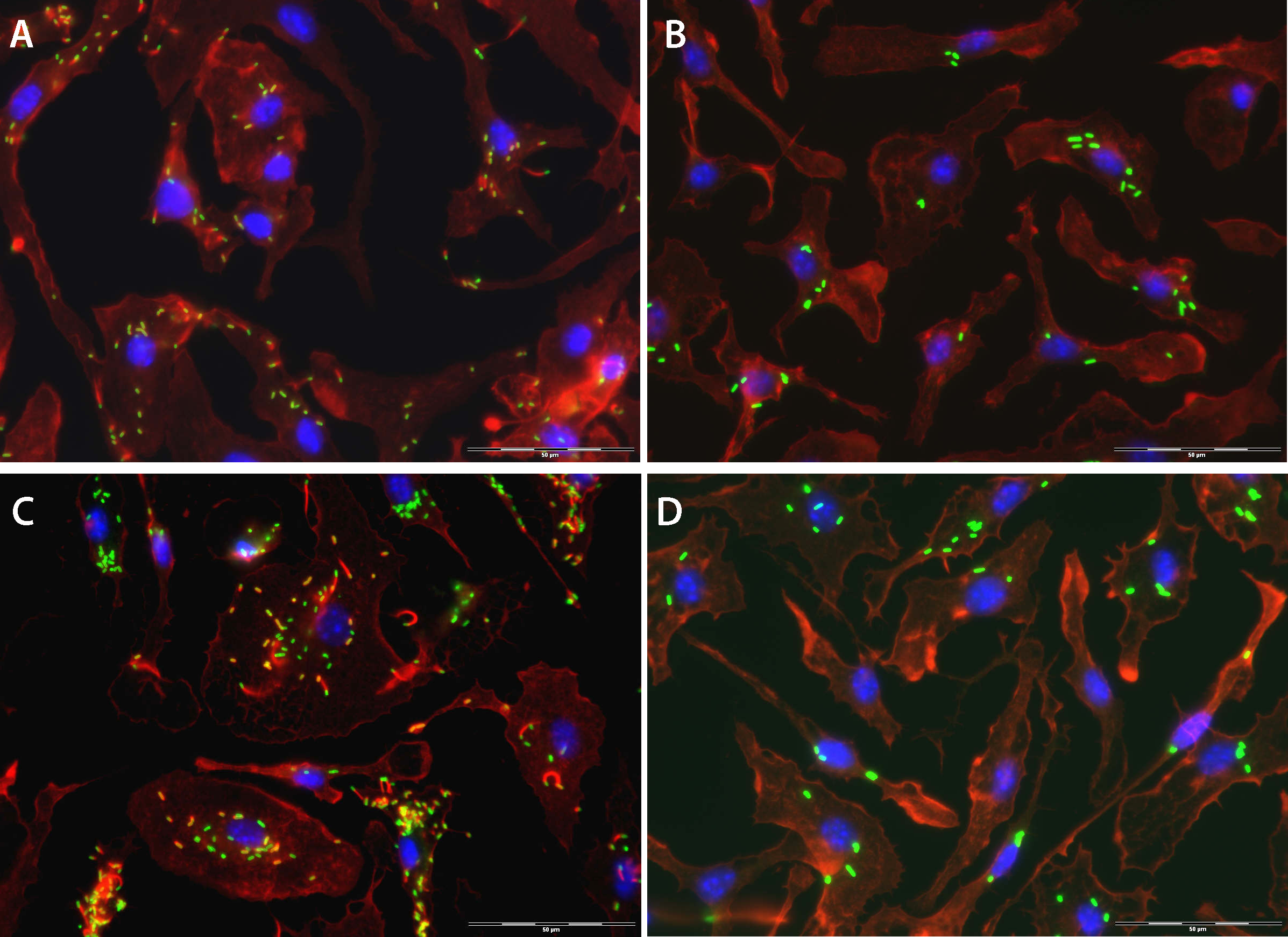

Supplement: Figure S2 — Immunofluorescence staining of macrophages infected with L. monocytogenes ( Lm ). Bone marrow derived macrophages (BMDMs) were infected (MOI 10) with Lm (A and C) or the LLO-deficient mutant Δhly (B and D) for 3 h (A and B) and 6 h (C and D). Cells were fixed, permeabilized and stained with TRITC-phalloidin antibody against filamentous actin (red), anti-Listeria primary antibody with FITC-conjugated goat anti-rabbit secondary antibody, to detect bacteria (green). Cell nuclei were stained with DAPI (blue). Immunomicrographs were taken with a 40× objective. (TIF) [file pone.0027435.s002.tif]

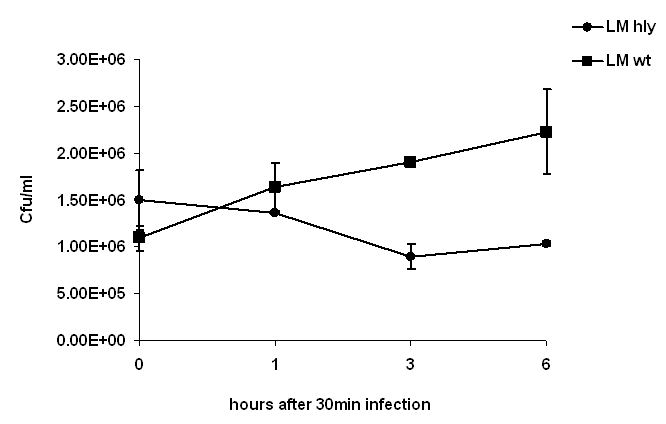

Supplement: Figure S3 — Growth curves of L. monocytogenes ( Lm ) wild type (wt) and Δhly mutant in macrophages. Cells were infected for 30 min and samples were collected at indicated timepoints post infection. Cells were lysed in 0.1% Triton-X100 in H2O and serial dilutions (10−2–10−6) were plated on blood agar plates overnight. Colony forming units were counted the next day and cfu/ml were plotted from 2–3 replicates. (TIF) [file pone.0027435.s003.tif]

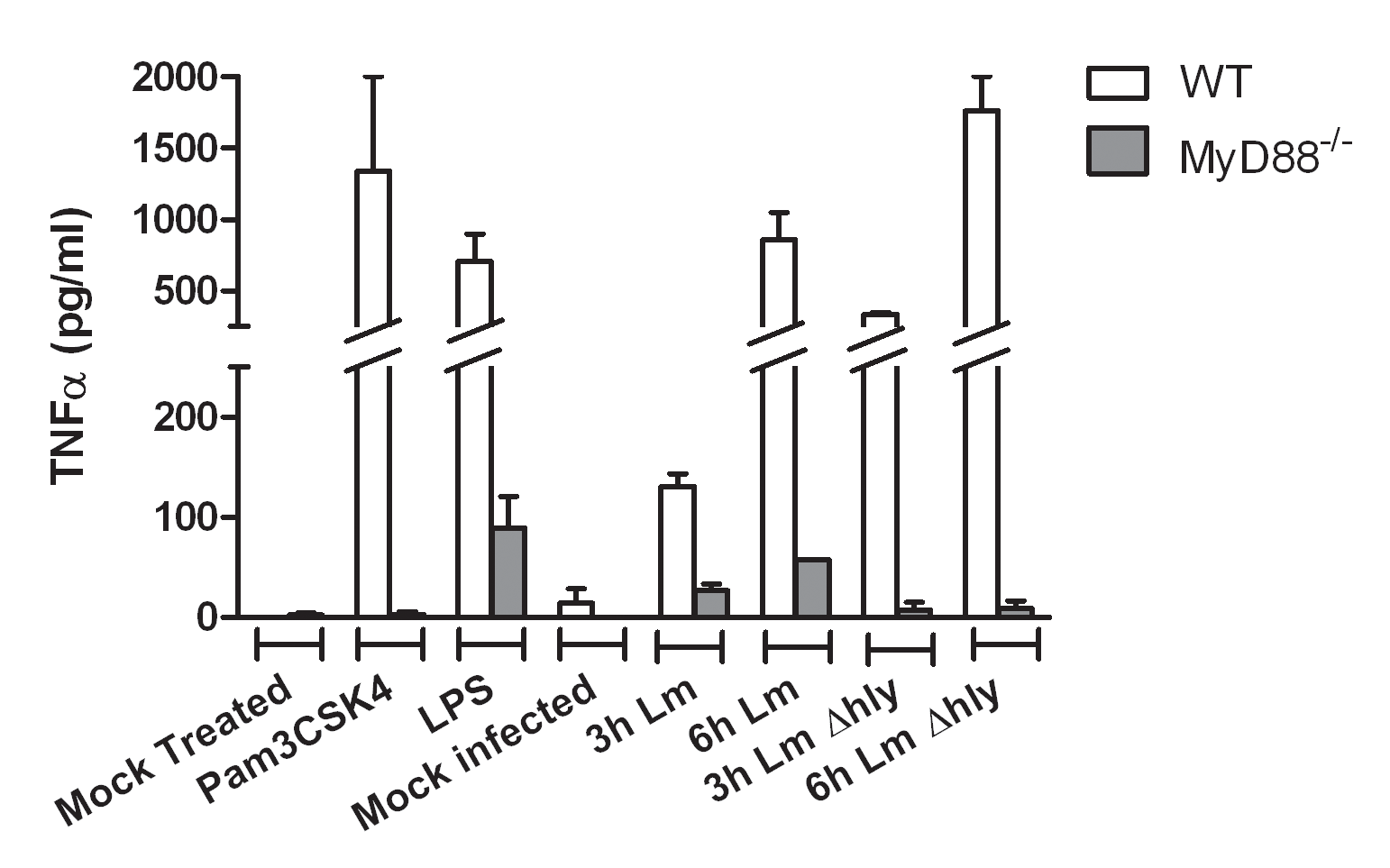

Supplement: Figure S4 — TNF production in wild type (WT) and MyD88−/− primary macrophages. Cells were treated for 6 h with 2 µg/ml Pam3CSK4 or 1 µg/ml LPS; or infected (MOI 10) for 3 h and 6 h with L. monocytogenes (Lm) or the LLO-deficient mutant Δhly (Lm Δhly). TNF (pg/ml) production was measured in the culture supernatant collected from 1×10^6^ cells/ml by ELISA. Data represents the mean values ± SEM from two biological replicates. (TIF) [file pone.0027435.s004.tif]

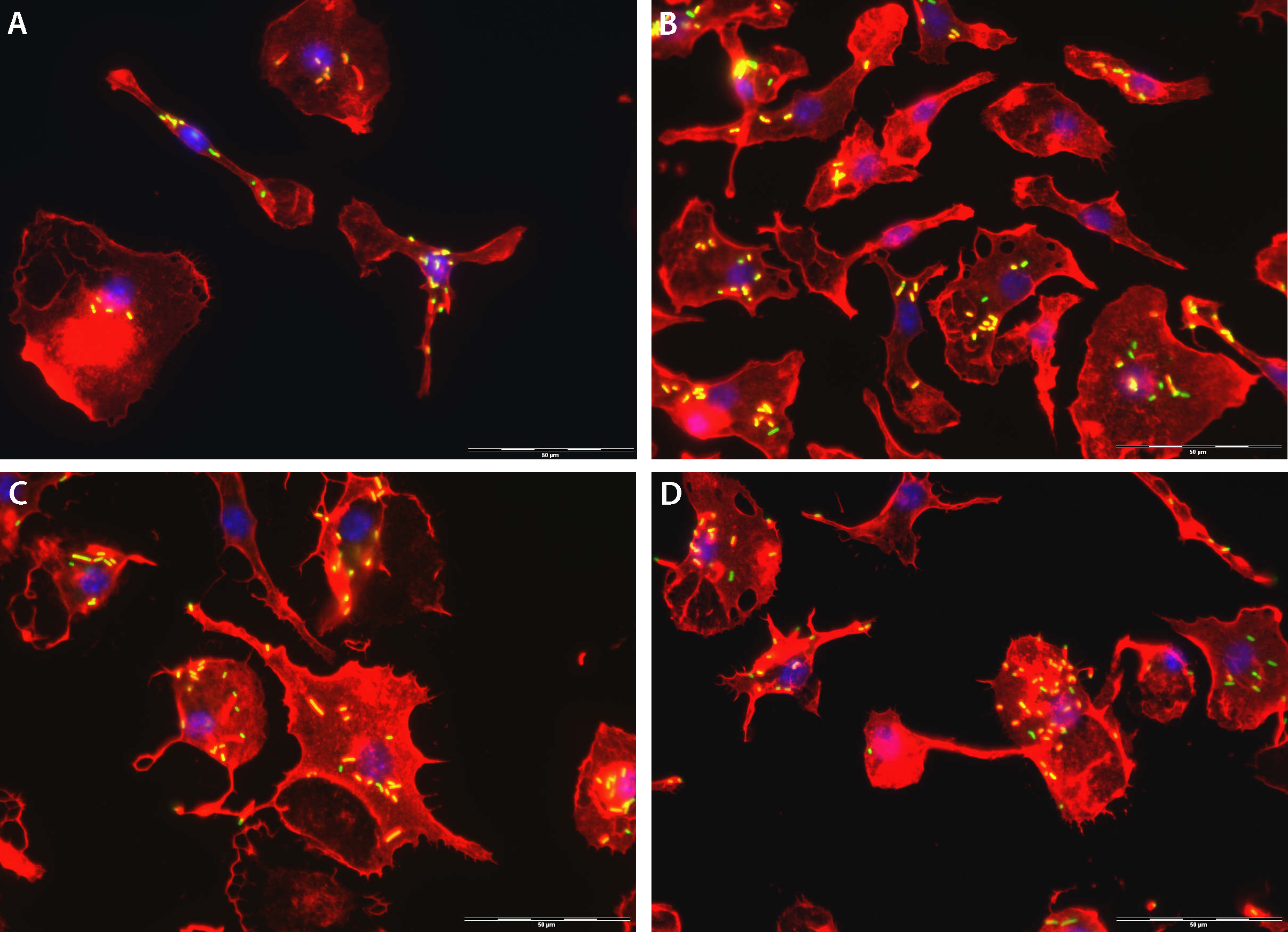

Supplement: Figure S5 — Immunofluorescence staining of macrophages infected with L. monocytogenes ( Lm ). Wild type (WT; A and C) or MyD88−/− (B and D) bone marrow derived macrophages (BMDMs) were infected with Lm MOI 10, for 3 h (A and B) or 6 h (C and D). Cells were then fixed, permeabilized and stained with TRITC-phalloidin antibody against filamentous actin (red), anti-Listeria primary antibody with FITC-conjugated goat anti-rabbit secondary antibody, to detect bacteria (green). Cell nuclei were stained with DAPI (blue). Immunomicrographs were taken with a 40× objective. (TIF) [file pone.0027435.s005.tif]

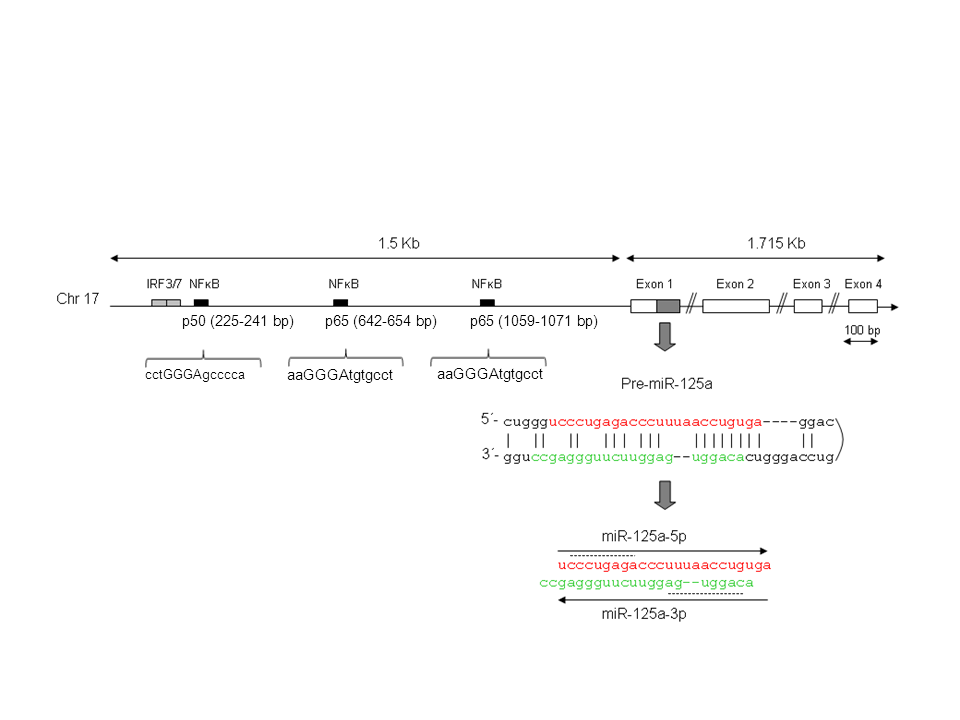

Supplement: Figure S6 — Schematic diagram of miR-125a genomic locus on mouse chromosome 17. Putative binding sites of NF-κB and IRF3/7 are shown (boxes) within the 1.5 kb upstream region of the pri-miR-125a transcriptional start site. Pre-miR-125a matures in the cytoplasm giving rise to mature miR-125a-5p and miR-125a-3p. (TIF) [file pone.0027435.s006.tif]

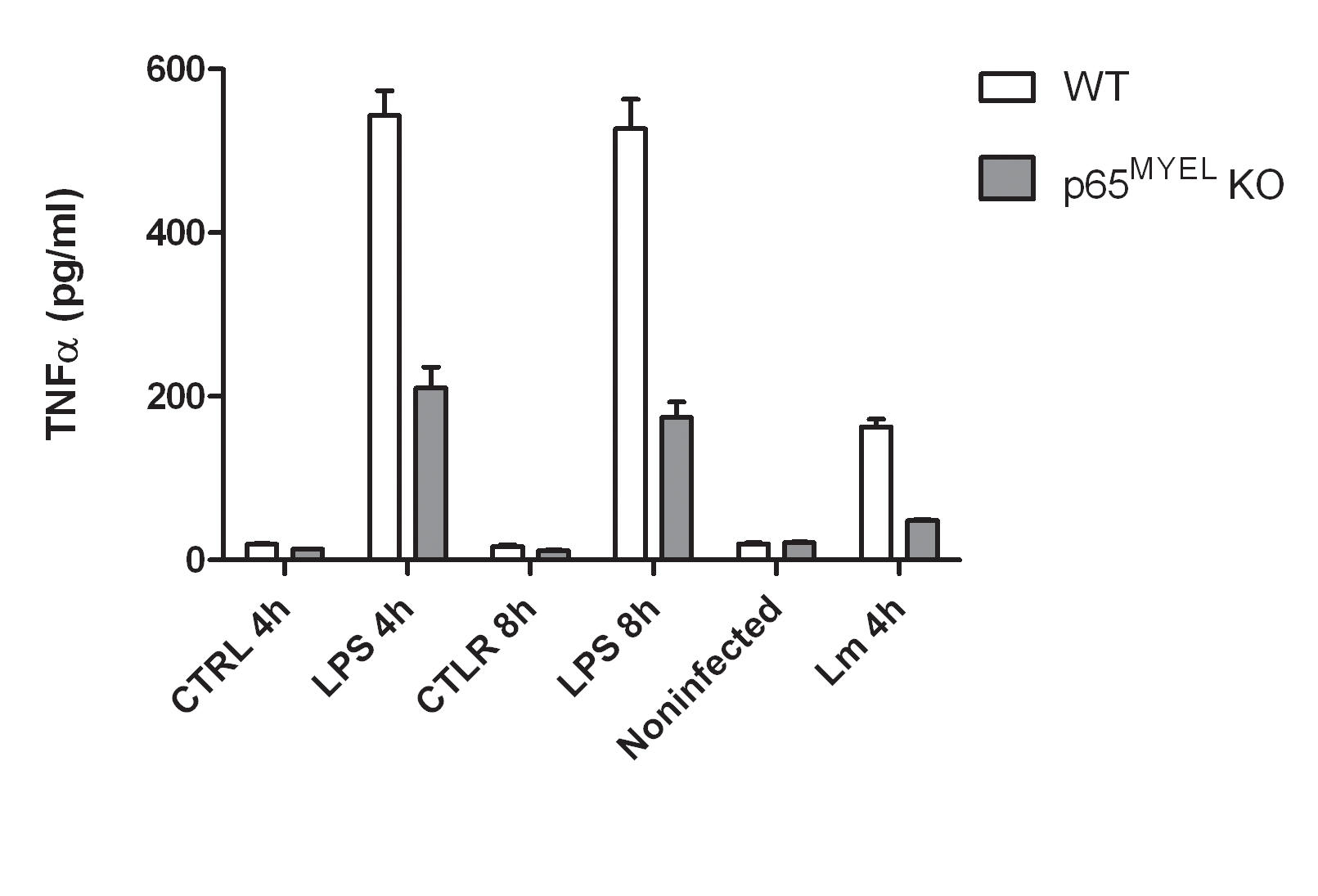

Supplement: Figure S7 — TNF production in wild type and p65MYELKO macrophages. WT (n = 2) and p65MYELKO (n = 2) bone marrow derived macrophages (BMDMs) were infected with L. monocytogenes (Lm) MOI 1 for 4 h, or treated with 100 ng/ml LPS for 4 h and 8 h. TNF (pg/ml) production was measured in the culture supernatant collected from 1×10^6^ cells/ml by ELISA. Data represents the mean values ± SEM from one experiment. (TIF) [file pone.0027435.s007.tif]

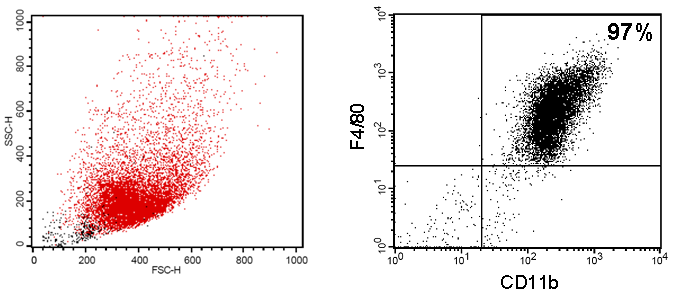

Supplement: Figure S8 — Characterisation of differentiated bone marrow derived macrophages (BMDMs) by FACS. BMDMs from days 7–8 of differentiation were stained with F4/80 (eBiosciences) and/or CD11b (BD) antibodies and analysed at the FACS Calibur. Quadrant statistics was performed using Cell Quest (BD). (TIF) [file pone.0027435.s008.tif]
