# Supplementary material for: Listeria monocytogenes Infection in Macrophages Induces Vacuolar-Dependent Host miRNA Response
Source: PLoS One. 2011 Nov 17;6(11):e27435. doi: 10.1371/journal.pone.0027435 (PMC3219661; doi:10.1371/journal.pone.0027435)
Supplement: Table S1 — Predicted and confirmed miRNA targets selected on the basis of their potential or known involvement in the anti-bacterial immune response of macrophages. Putative targets were predicted using TargetScan 5.1. Confirmed targets were selected from the cited studies. (DOCX) [file pone.0027435.s009.docx]

| **miRNA** | **Target mRNA** | **Function** |
| --- | --- | --- |
| **miR-146a** | Traf6 ^[1-3]^ | TNF receptor-associated factor 6; E3 ubiquitin-protein ligase, mediates receptor signal transduction of the TNF and TLR/IL-1 family members |
|  | IRAK1 and IRAK2 ^[1-3]^ | Interleukin-1 receptor-associated kinase (IRAK) 1 and 2; serine/threonine kinases associated with TLR/IL-1 receptor signalling |
|  | Smad4 | Transcriptional modulator downstream of TGFβ signalling |
|  | CSF1R | Colony stimulating factor 1 receptor; mediates differentiation and function of macrophages |
| **miR-155** | FADD ^[4]^ | Adaptor molecule that recruits caspase-8 or caspase-10 to the activated Fas (CD95), TRAILR or TNFR-1 receptors |
|  | RIPK1 ^[4]^ | A serine/threonine kinase that mediates both necrosis and NF-κB activation |
|  | Smad2 ^[5]^ | Transcriptional modulator downstream of TGFβ signalling pathway |
|  | SHIP-1 ^[6-8]^ | SH2 domain containing inositol-5-phosphatase; involved in FcγR signalling and phagocytosis |
|  | SOCS1 ^[9]^ | Suppressor of cytokine signalling; Negative regulator of TLR4 |
|  | Lamp2 | Lysosomal-associated membrane protein 2 |
|  | Card11 | Caspase recruitment domain family, member 11;NF-κB activator together with Bcl10 |
|  | Socs6 | Suppressor of cytokine signaling |
|  | CSF1R | Colony stimulating factor 1 receptor; mediates differentiation and function of macrophages |
|  | Septin11 | Depletion increases Listeria invasion in epithelial cells |
| **miR-125a-3p** | IL-1 R1 | IL-1 Receptor 1 signalling |
|  | Septin11 | Depletion increases Listeria invasion in epithelial cells |
| **miR-125a-5p** | IL-6 R | IL-6 Receptor signalling |
|  | M6PR | Mannose-6-phosphate receptor |
|  | Smad4 | Transcription factor; TGFβ signalling pathway |
| **miR-149** | Tirap | TIR domain containing adaptor protein |
|  | Smad2 | Transcription factor; TGFβ signalling pathway |
|  | SOCS6 | Suppressor of cytokine signalling |
|  | Septin9 | Interacting partner of septin11 |
| Abbreviations: Bcl, B-cell lymphoma; FADD, Fas-Associated protein with Death Domain; FcγR, Fc-gamma receptors; NF-κB, nuclear factor 'kappa-light-chain-enhancer' of activated B-cells; RIPK1, Receptor-interacting serine/threonine-protein kinase 1; TIR, Toll/IL (interleukin)-1 receptor] superfamily; Tirap, TIR domain containing adaptor protein; TLR, toll-like receptor; TNFR, tumour necrosis factor (TNF) receptor; TGFβ, Transforming growth factor (TGF)-beta; TRAIL, TNF-related apoptosis-inducing ligand.  Citations: 1. Taganov et al (2006) Proc Natl Acad Sci U S A 103(33):12481-12486; 2. Hou et (2009) J Immunol 183(3):2150-2158; 3. Nahid et al (2009) J Biol Chem.11;284(50):34590-9; 4. Tili et al (2007) J Immunol 179(8):5082-5089; 5. Xiao et al (2009) J Infect Dis 200(6):916-925 ; 6. O'Connell et al (2007) Proc Natl Acad Sci U S A 104(5):1604-1609; 7. Cremer et al (2009) PLoS One 30;4(12):e8508; 8. Costinean et al (2009) Blood 13;114(7):1374-82; 9. Lu et al (2009) Immunity. 30(1):80-91. | | |
